# Releasing the brakes: the role of immune checkpoint inhibitors in laryngeal cancer

**DOI:** 10.37349/etat.2025.1002292

**Published:** 2025-02-17

**Authors:** Michail Athanasopoulos, Pinelopi Samara, Georgios Agrogiannis, Ioannis Athanasopoulos, Nikolaos Kavantzas, Efthymios Kyrodimos, Nicholas S. Mastronikolis

**Affiliations:** University of Salford, UK; ^1^Department of Otolaryngology, University Hospital of Patras, 26504 Patras, Greece; ^2^Children’s Oncology Unit “Marianna V. Vardinoyannis-ELPIDA”, Aghia Sophia Children’s Hospital, 11527 Athens, Greece; ^3^1st Department of Pathology, School of Medicine, National and Kapodistrian University of Athens, 11527 Athens, Greece; ^4^Department of Audiology, Otology, Neurotology & Cochlear Implant Unit, Athens Pediatric Center, 15125 Athens, Greece; ^5^1st Department of Otolaryngology, Hippocration Hospital, National and Kapodistrian University of Athens, 11527 Athens, Greece

**Keywords:** Laryngeal cancer, immune checkpoint inhibitors, nivolumab, pembrolizumab, PD-1/PD-L1, CTLA-4, clinical trials, immune-related adverse events

## Abstract

Laryngeal cancer, a subtype of head and neck cancer, poses significant challenges due to its profound impact on essential functions such as speech and swallowing and poor survival rates in advanced stages. Traditional treatments—surgery, radiotherapy, and chemotherapy—are often associated with high morbidity and substantial recurrence rates, emphasizing the urgent need for novel therapeutic approaches. Immune checkpoint inhibitors (ICIs) have revolutionized oncology by countering tumor-induced immune evasion, restoring immune surveillance, and activating T-cell responses against cancer. This review examines the role of ICIs in laryngeal cancer management, with a focus on pembrolizumab and nivolumab (anti-PD-1 agents), which are clinically established, as well as investigational therapies such as dostarlimab (anti-PD-1), atezolizumab (anti-PD-L1), and ipilimumab (anti-CTLA-4). Pembrolizumab, in combination with platinum-based chemotherapy and 5-fluorouracil, is approved as a first-line treatment for recurrent or metastatic head and neck squamous cell carcinoma (HNSCC), based on evidence from the Keynote-048 trial. This pivotal trial demonstrated significant overall survival (OS) benefits over the cetuximab-based standard regimen. Similarly, nivolumab showed improved OS in the CheckMate-141 trial, supporting its approval as a second-line therapy for patients with platinum-refractory disease. ICIs have shown durable survival benefits and a more manageable toxicity profile compared to traditional chemotherapy. Immune-related adverse events are generally mild and controllable; however, in some cases, they can become severe and even life-threatening. Furthermore, ICIs are being investigated in combination with radiotherapy, as well as in neoadjuvant and adjuvant settings, where preliminary findings suggest these approaches may enhance efficacy, preserve organ function, and overcome resistance to conventional treatments. The integration of ICIs into multimodal treatment strategies holds promise for transforming the therapeutic landscape of advanced laryngeal cancer. This review synthesizes current evidence, highlights ongoing research, and explores strategies to enhance survival and quality of life for patients facing this challenging malignancy.

## Introduction

Laryngeal cancer, a subtype of head and neck cancer (HNC) that arises from the tissues of the larynx, represents a significant global health challenge. Predominantly characterized by squamous cell carcinoma, this malignancy affected 184,615 individuals worldwide in 2020, resulting in over 99,000 deaths, with significant regional variations in incidence and mortality [1]. In the United States, an estimated 12,380 new cases of laryngeal cancer were diagnosed in 2023, with approximately 4,000 deaths attributed to the disease [2]. Laryngeal cancer primarily affects men over the age of 55 and is strongly linked to risk factors such as smoking, alcohol consumption [3], and human papillomavirus (HPV) infection [4].

Traditional treatment modalities, including surgery, radiotherapy, and chemotherapy, are tailored to the tumor’s stage and location but are associated with high morbidity and recurrence rates. Despite advancements, survival outcomes for advanced disease remain poor, with a 5-year survival rate of approximately 60%, which drops significantly in metastatic cases [2]. While surgical interventions like partial and total laryngectomy can be effective, they often lead to functional consequences including voice loss and airway compromise [5]. Likewise, radiotherapy and chemotherapy are essential for managing advanced cases but are accompanied by adverse effects, such as mucositis, xerostomia, and systemic toxicities, which can greatly impact the patient’s quality of life [6].

Immune checkpoint inhibitors (ICIs) have revolutionized the therapeutic landscape for cancer, offering new hope for improved survival. ICIs function by blocking checkpoint molecules, such as PD-1 (programmed cell death-1), PD-L1 (programmed death-ligand 1), and CTLA-4 (cytotoxic T-lymphocyte-associated protein 4), which tumors exploit to evade immune detection [7]. By targeting these pathways, ICIs help reactivate the immune system, enabling it to recognize and attack cancer cells. Pembrolizumab (Keytruda, Merck, Kenilworth, NJ) and nivolumab (Opdivo, Bristol Meyer Squibb, New York, NY), both anti-PD-1 agents, have become well-established in clinical practice [8]. Pembrolizumab, in combination with platinum-based chemotherapy and 5-fluorouracil (5FU), is approved as a first-line treatment for recurrent or metastatic head and neck squamous cell carcinoma (HNSCC) [9]. Nivolumab is approved as a second-line treatment for laryngeal cancer resistant to platinum-based chemotherapy [10]. Beyond these approved agents, several ICIs are under investigation for laryngeal cancer, including dostarlimab (anti-PD-1), ipilimumab and tremelimumab (anti-CTLA-4), atezolizumab and durvalumab (anti-PD-L1), and lirilumab (anti-killer cell immunoglobulin-like receptor) [11]. These agents aim to overcome the immune evasion mechanisms employed by tumors, providing additional options in the ongoing fight against this malignancy.

This review explores the role of ICIs in the management of laryngeal cancer, with a focus on both established treatments and investigational agents. It examines their mechanisms of action, clinical efficacy, safety profiles, and potential to address the limitations of existing therapies. By integrating ICIs into current treatment paradigms, there is hope to redefine outcomes, enhancing survival and quality of life for patients facing this challenging malignancy.

## Mechanism of action

### Immune evasion by tumors

Tumors possess sophisticated mechanisms to evade immune detection, enabling their growth and metastasis [12]. A primary strategy involves exploiting immune checkpoint pathways—natural regulatory systems that prevent overactivation of the immune response to maintain self-tolerance and prevent autoimmunity. By upregulating checkpoint proteins, tumors suppress T-cell activity, which is critical in recognizing and eliminating cancer cells. This immune suppression allows tumors to grow unchecked, evading destruction by the body’s immune system [13].

### Checkpoint inhibition mechanism

**Figure 1:**
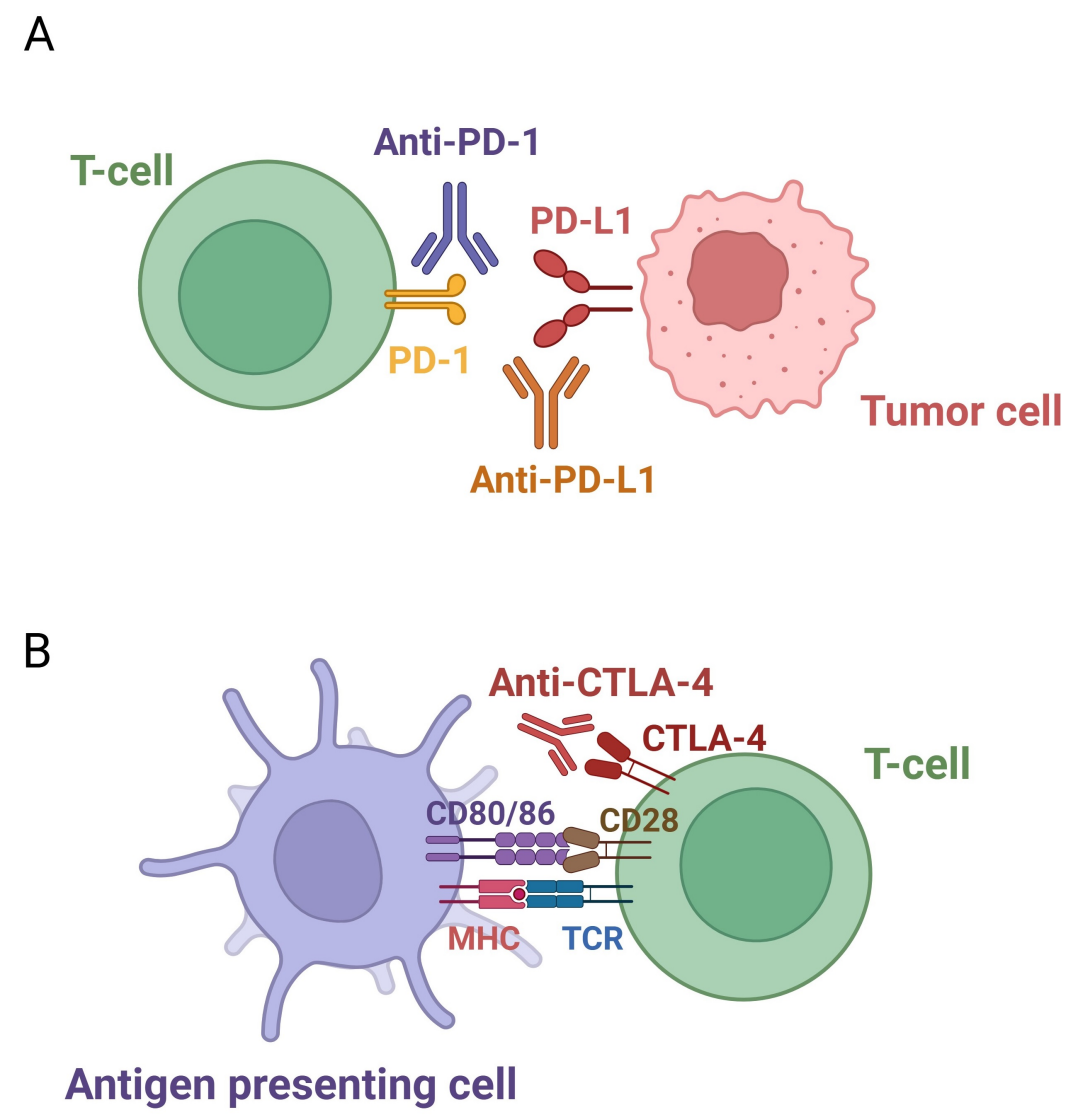
**Pathways of immune checkpoint inhibition.** (**A**) PD-1/PD-L1 pathway: PD-1 on T-cells binds to PD-L1 on tumor or immune cells, suppressing T-cell activity. ICIs targeting either PD-1 or PD-L1 block this interaction, thereby restoring T-cell function. (**B**) CTLA-4 pathway: CTLA-4 on T-cells competes with CD28 for binding to CD80/CD86 on antigen-presenting cells, dampening T-cell activation. ICIs block CTLA-4, thereby enhancing immune responses against tumors. PD-1/PD-L1: programmed cell death-1/programmed death-ligand 1; ICIs: immune checkpoint inhibitors; CTLA-4: cytotoxic T-lymphocyte-associated protein 4. Created in BioRender. Sam, P. (2025) https://BioRender.com/a93a062

ICIs are a groundbreaking class of immunotherapies designed to counteract tumor-induced immune suppression. By blocking checkpoint proteins such as PD-1, PD-L1, and CTLA-4, ICIs release the “brakes” on the immune system, reactivating T-cells and enhancing their ability to effectively target and destroy cancer cells [14] (Figure 1).

#### PD-1/PD-L1, and CTLA-4 pathways

##### PD-1/PD-L1 pathway

PD-1 is a checkpoint protein expressed on T-cells that binds to its ligands, PD-L1 and PD-L2. These ligands are frequently overexpressed by tumor cells and within the tumor microenvironment. The interaction between PD-1 and PD-L1 delivers inhibitory signals that suppress T-cell activity, allowing tumors to evade immune detection. ICIs targeting the PD-1/PD-L1 axis block this interaction, restoring T-cell function and facilitating a stronger anti-tumor response [15] (Figure 1A).

##### CTLA-4 pathway

CTLA-4 is another checkpoint protein expressed on T-cells, acting as a critical regulator of T-cell activation. It competes with CD28, a stimulatory receptor on T-cells, for binding to the ligands CD80 and CD86 on antigen-presenting cells. When CTLA-4 binds to these ligands, it transmits inhibitory signals that reduce T-cell proliferation and suppress immune responses. Tumors exploit this mechanism by inducing CTLA-4 expression, further dampening the immune system. ICIs such as ipilimumab and tremelimumab target CTLA-4 by blocking its interaction with CD80 and CD86, thereby preventing inhibitory signaling. This enables T-cells to remain active and effectively attack cancer cells [16] (Figure 1B).

## Unique features of the larynx and their implications for ICI therapy in laryngeal cancer

Among head and neck malignancies, laryngeal cancer exemplifies the complex interplay between environmental exposures, chronic inflammation, and immune modulation within a highly specialized anatomical site. Its distinct tumor microenvironment—characterized by unique vascular, lymphatic, and immunological features—serves as a valuable model for investigating the mechanisms and therapeutic challenges of ICIs. Furthermore, the larynx’s critical roles in phonation and airway protection require approaches that balance therapeutic effectiveness with the preservation of vital functions, thereby optimizing patients’ quality of life [17].

The larynx is a multifunctional organ critical for phonation, respiration, and airway protection [18], continuously exposed to inhaled irritants such as smoke, pollutants, and pathogens. This chronic exposure predisposes the larynx to inflammation and epithelial damage, both of which are significant drivers of carcinogenesis [19]. These factors, along with the mechanical stresses associated with voice production, influence tumor dynamics and complicate the tumor microenvironment. Laryngeal cancers often arise in areas that are highly vascularized and innervated, which can hinder immune cell infiltration and the delivery of therapies [20]. Moreover, the laryngeal mucosa acts as a frontline barrier against external antigens, hosting a diverse array of immune cells [21]. Continuous activation of these defenses by environmental exposures may result in chronic inflammation and immune exhaustion, frequently marked by upregulation of immune checkpoint molecules such as PD-1 and PD-L1 [22]. This state of immune exhaustion creates an ideal setting for the application of ICIs.

The tumor microenvironment in laryngeal cancer reflects a dynamic interplay among tumor cells, stromal components, and immune elements. Chronic inflammation and exposure to environmental toxins create an immunosuppressive milieu, dominated by regulatory T-cells and myeloid-derived suppressor cells. Additionally, the unique lymphatic and vascular architecture of the larynx may significantly influence immune cell infiltration and, consequently, the efficacy of ICIs [23]. The critical roles of the larynx in voice production and airway protection introduce unique challenges in managing immune-related adverse events (irAEs). Inflammation or fibrosis resulting from immunotherapy could compromise these essential functions [24], highlighting the need for treatment strategies that balance immune modulation with functional preservation. The incorporation of ICIs into laryngeal cancer treatment represents a transformative step, offering new opportunities for personalized therapeutic strategies. Continued research is vital to harness the unique immunological landscape of the larynx while addressing the functional considerations critical to improving patient outcomes.

## Clinical trials in ICIs for HNC, including laryngeal cancer

The exploration of ICIs in the treatment of laryngeal cancer has gained significant momentum through numerous clinical trials, reflecting the growing interest in harnessing the potential of immunotherapy. These trials aim to assess the efficacy, safety, and role of ICIs in overcoming the limitations of conventional therapies [25]. In patients with advanced laryngeal cancer, ICIs have shown promise in improving overall survival (OS) and progression-free survival (PFS), addressing key challenges such as treatment resistance and recurrence.

As of January 2025, a query on ClinicalTrials.gov using the search terms “laryngeal cancer” and “immune checkpoint inhibitors” identified 35 studies at various stages of progress [26]. These include 15 trials currently recruiting participants, 8 active but no longer recruiting, 8 completed, 1 not yet recruiting, 1 suspended, 1 with unknown status, and 1 terminated. The study with unknown status (NCT05551767) has exceeded its completion date, with no status update in over two years. The terminated study (NCT03854032), which investigated the combination of nivolumab with the IDO1 inhibitor BMS-986205, was discontinued due to safety concerns related to toxicity [27]. The active trials expected to yield results are presented in Table 1 [26].

**Table 1:** Active clinical trials for laryngeal cancer with immune checkpoint inhibitors (ICIs) that are no longer recruiting [26]

**Trial identifier**	**Number of cancer patients**	**Intervention/Treatment regimen**	**Setting**	**Primary outcome(s)**	**Secondary outcome(s)**
NCT04943445	43 laryngeals	Pembrolizumab-based therapy	Neoadjuvant (organ preservation)	Two-year laryngectomy-free survival	Two-year larynx dysfunction-free survival (alive without local recurrence or laryngoesophageal dysfunction)
NCT04030455	27 laryngeals	Cisplatin, docetaxel, pembrolizumab	Neoadjuvant	Disease control rate, pathological complete response rate	Incidence of adverse events, laryngeal preservation rate, relapse-free survival, overall survival, patient-reported quality of life
NCT04590963	370 head and neck (laryngeal unspecified)	Monalizumab, cetuximab	Recurrent/Metastatic	Overall survival in HPV-unrelated analysis set	Overall survival in full analysis set, progression-free survival, duration of response
NCT02586207	59 head and neck (laryngeal unspecified)	Pembrolizumab + chemoradiotherapy (CRT)	Neoadjuvant	Safety & tolerability	Efficacy, response rate
NCT03082534	78 head and neck (laryngeal unspecified)	Pembrolizumab + cetuximab	Recurrent/Metastatic	Clinical efficacy	Progression-free survival, overall survival, duration of response
NCT04576091	7 head and neck (laryngeal unspecified)	Pembrolizumab + radiation + BAY 1895344 (elimusertib)	Recurrent	Safety & tolerability	Incidence of adverse events, locoregional control, progression-free survival, overall response rate, 1-year overall survival, quality of life
NCT03370276	95 head and neck (laryngeal unspecified)	Cetuximab + nivolumab	Recurrent/Metastatic	Phase I: maximum tolerated dose, Phase II: overall survival	Overall response rate, progression-free survival, treatment-related adverse events
NCT03468218	36 head and neck (laryngeal unspecified)	Pembrolizumab + cabozantinib	Recurrent/Metastatic	Overall response rate	Progression-free survival

Not all studies involving ICIs have yielded positive outcomes. The phase III JAVELIN trial, which assessed the addition of avelumab to standard chemoradiotherapy in patients with locally advanced HNSCC, failed to achieve its primary goal of prolonging PFS [28]. In fact, PFS was similar between the avelumab and placebo groups, with the placebo group showing a slightly better hazard ratio. This outcome may be attributed to the addition of avelumab compromising the benefits of cisplatin and radiation, possibly through immune microenvironment changes or T-cell depletion. Despite these disappointing results, the trial provided valuable insights into the complexities of integrating immunotherapy with standard treatment modalities. It underscored the critical roles of treatment timing, patient selection, and the need to optimize combination therapies to enhance clinical outcomes. These findings highlight the importance of tailoring treatments to individual patient profiles, ensuring maximal benefit while minimizing potential interference with conventional therapies.

Nevertheless, ongoing clinical trials continue to explore the therapeutic potential and clinical applications of ICIs in laryngeal cancer. These studies primarily focus on refining ICI use through innovative combination therapies, identifying predictive biomarkers to better stratify patients, and investigating their utility in early-stage disease and adjuvant settings. Emerging evidence suggests that ICIs, whether used as monotherapy or in conjunction with other treatments, have the potential to significantly enhance treatment effectiveness. This progress has reinvigorated optimism, particularly for patients with advanced or recurrent laryngeal cancer, who often have limited treatment options.

The approval of ICIs, namely nivolumab and pembrolizumab, for the treatment of laryngeal cancer has been supported by the following clinical trials demonstrating their efficacy in advanced HNSCC. The CheckMate 141 trial (NCT02105636), conducted by Ferris et al. [29] evaluated nivolumab in 361 patients with platinum-refractory recurrent or metastatic HNSCC, including 49 patients with laryngeal cancer. Nivolumab achieved a median OS of 7.5 months compared to 5.1 months with standard chemotherapy, with a 1-year survival rate of 36% vs. 16.6%. Additionally, nivolumab was associated with fewer grade 3–4 adverse events (13.1% vs. 35.1%) and better preservation of quality of life, establishing it as a viable second-line treatment option for advanced HNSCC, including laryngeal cancer [29].

The Keynote-012 trial (NCT01848834), led by Seiwert et al. [30] investigated pembrolizumab in 60 patients with PD-L1-positive recurrent or metastatic HNSCC, including two with laryngeal cancer. Pembrolizumab demonstrated an overall response rate of 18%, with higher efficacy observed in HPV-positive patients (25%) [30]. These promising results were further expanded upon in the Keynote-048 trial (NCT02358031), which established pembrolizumab as a first-line treatment for recurrent or metastatic HNSCC, including laryngeal cancer, both as monotherapy and in combination with chemotherapy. In this trial, pembrolizumab monotherapy significantly improved OS in patients with a combined positive score (CPS) ≥ 20 for PD-L1 expression, while the combination of pembrolizumab with chemotherapy demonstrated superior OS across all subgroups. Notably, this combination also showed reduced toxicity compared to standard treatments, further enhancing its clinical value [31, 32].

Locally advanced laryngeal cancers have also benefited from novel ICI-based combination therapies. Frankart et al. [33] reported an impressive 100% laryngeal preservation rate with the combination of pembrolizumab and chemoradiation in patients with stage III/IV laryngeal cancer. The NRG-HN003 study further demonstrated the feasibility and safety of adjuvant pembrolizumab combined with cisplatin and intensity-modulated radiation therapy for high-risk HPV-negative HNSCC [34]. Similarly, Uppaluri et al. [35] observed significant reductions in relapse rates using neoadjuvant and adjuvant pembrolizumab in resectable HNSCC. In their phase II trial, which included 10 patients with laryngeal cancer, high-risk cases showed a 1-year relapse rate of 16.7%, markedly lower than the historical rate of 35%. These findings suggest pembrolizumab’s potential role in reducing relapse rates in high-risk resectable HNSCC. Additionally, Hanna et al. [36] explored dual checkpoint inhibition with nivolumab and lirilumab, achieving a 43% pathologic response rate in resectable HNSCC, including 9 patients with laryngeal cancer.

Therapeutic strategies, including dual checkpoint inhibition and radioimmunotherapy, have also shown promise. In a phase I trial conducted by Ferris et al. [37] in 2022, the combination of cetuximab, radiotherapy, and ipilimumab was evaluated in patients with locally advanced HNSCC. The study reported favorable tolerability and promising efficacy, with a 3-year disease-free survival (DFS) rate of 72%, suggesting the potential of this combination for improving long-term disease control [37]. Similarly, Johnson et al. [38] reported a 3-year PFS rate of 74% using nivolumab and ipilimumab in conjunction with radiotherapy. This regimen, tested in 24 patients, including 6 with laryngeal cancer, demonstrated substantial efficacy but was associated with a higher incidence of adverse events.

Three noteworthy studies, two ongoing and one recruiting, are exploring advanced therapeutic approaches for HNC [26]. The first is a phase II trial (NCT04030455) investigating cisplatin, docetaxel, and pembrolizumab in stage II–III laryngeal cancer. This study evaluates clinical response rates after two cycles, complete pathological response after four cycles, and secondary outcomes such as safety, laryngeal preservation at two years, survival rates, and patient-reported quality of life. Responders may continue pembrolizumab monotherapy, with follow-ups lasting up to two years. The second is a phase II clinical trial (NCT04313504) evaluating the PD-1 inhibitor dostarlimab in combination with the PARP inhibitor niraparib for recurrent or metastatic HNSCC. This trial measures response rates, PFS, and OS, aiming to provide insights into the potential of this novel combination in advanced cases. The third is a phase II/III trial (NCT05063552) currently recruiting to evaluate adding bevacizumab to standard chemotherapy (cisplatin, carboplatin, or docetaxel with cetuximab) or combining bevacizumab with atezolizumab in recurrent or metastatic HNC. The primary endpoints include PFS and OS, while secondary objectives assess outcomes in patients with high PD-L1 expression (CPS ≥ 20), treatment-related toxicity, and imaging biomarkers. This trial seeks to determine whether these regimens can surpass the current standard of care in improving survival and disease control [26]. By leveraging data from all these trials, the potential of ICIs to reshape treatment paradigms for both laryngeal and other HNCs continues to grow.

## Key biomarkers for predicting response to ICIs in laryngeal and other HNCs

Biomarkers play a crucial role in predicting the response to ICIs in laryngeal and HNCs, enabling the development of personalized treatment strategies by identifying patients most likely to benefit from these therapies. Key biomarkers include PD-L1 expression and tumor mutational burden (TMB) [39]. Higher PD-L1 expression, particularly with a CPS of ≥ 20%, is associated with better outcomes for patients treated with PD-1/PD-L1 inhibitors, aiding in patient selection [40]. A recent meta-analysis highlighted that high TMB, which reflects the number of mutations in a tumor, is a predictive biomarker for improved response to ICIs in HNSCC. Patients with high TMB demonstrated a significantly better response rate and survival advantage, as these tumors are more likely to present neoantigens that can trigger immune responses [41]. However, there are challenges in applying these biomarkers clinically. PD-L1 expression is heterogeneous within tumors and across different sites, which can impact its predictive accuracy [40]. Additionally, the variability of PD-L1 over time and the lack of standardized TMB testing protocols further complicate their clinical utility, underscoring the need for additional biomarkers to improve prediction and treatment outcomes [42].

Emerging biomarkers, such as microsatellite instability (MSI), T-cell exhaustion markers, and features of the tumor microenvironment, are being actively explored to refine patient selection and treatment strategies [43, 44]. MSI-high tumors, which are more immunogenic, often show better responses to ICIs. Markers such as lymphocyte activation gene-3, T-cell immunoglobulin and mucin-domain containing-3, and T-cell immunoreceptors with immunoglobulin and tyrosine-based inhibitory motifs are emerging as promising targets in HNC immunotherapy. These markers offer the potential to overcome T-cell dysfunction and resistance by addressing the mechanisms through which tumors evade immune surveillance [45]. Future research aims to develop a comprehensive multi-biomarker approach that combines PD-L1 expression, TMB, genetic profiling, immune cell infiltration, and tumor microenvironment features.

## Safety and adverse events of ICIs in patients with HNC

ICIs have significantly advanced cancer therapy, providing substantial efficacy in various malignancies, including laryngeal cancer. However, the heightened immune activation induced by ICIs is associated with a distinct spectrum of side effects, necessitating vigilant management to optimize patient outcomes [46]. The safety profiles of ICIs have been extensively evaluated in clinical trials, showing that these therapies are generally well-tolerated by most patients. Common adverse effects, such as fatigue, dermatological reactions (e.g., rash), and mild endocrine disorders, are typically transient and manageable with supportive care or dosage adjustments. These effects usually resolve upon discontinuation or modification of therapy.

More concerning are irAEs, which, while less frequent, can be severe and potentially life-threatening [47]. Common irAEs include colitis, hepatitis, pneumonitis, and endocrinopathies (Table 2). These conditions arise from the immune system’s attack on healthy tissues and often require prompt intervention. Despite the potential severity of irAEs, clinical evidence suggests their incidence is relatively low, and the therapeutic benefits of ICIs generally outweigh these risks, particularly in patients with advanced or refractory cancers. Early detection and appropriate management protocols have significantly improved the safety profile of ICIs, allowing most patients to continue therapy with minimal interruptions.

**Table 2:** Spectrum and effect sizes (any grade and grade 3 or higher) of immune checkpoint inhibitor (ICI)-related adverse events, categorized by organ system/type, as reported in a systematic review and meta-analysis by Dang et al. [48]

**Adverse event category**	**Specific adverse events**	Effect size (%)*, (any grade)	**Effect size (%), (grade 3 or higher)**
Fatigue	Ranging from mild to severe intensity	14.95	0.74
Dermatological reactions	Rash, pruritus (itching), vitiligo, erythema, dermatitis	Rash: 8.66; Pruritus: 7.5	Rash: 0.26; Pruritus: rare
Gastrointestinal issues	Diarrhea, colitis, nausea, vomiting, abdominal pain	Diarrhea: 6.77; Nausea: 5.57; Vomiting: 2.49; Colitis: 0.62	Diarrhea: 0.35; Nausea: very rare; Vomiting: very rare; Colitis: 0.34
Endocrinopathies	Hypothyroidism, hyperthyroidism, adrenal insufficiency, diabetes mellitus, hypophysitis	Hypothyroidism: 11.21; Hyperthyroidism: 3.29	Hypothyroidism: very rare; Hyperthyroidism: rare
Hepatotoxicity	Elevated liver enzymes, autoimmune hepatitis, jaundice	Hepatitis: 1.97; AST increased: 4.75; ALT increased: 3.48	Hepatitis: 1.13; AST increased: 0.65; ALT increased: 0.19
Pulmonary toxicity	Pneumonitis, interstitial lung disease	Pneumonitis: 2.92	Pneumonitis: 0.95
Renal toxicity	Nephritis, acute kidney injury	Nephritis: 0.51	Nephritis: 0.30
Neurological issues	Peripheral neuropathy, encephalitis, Guillain-Barre syndrome, myasthenia gravis	Neuropathy: 1.02	Neuropathy: 0.04
Hematological toxicity	Thrombocytopenia, pancytopenia or immune aplastic anemia, neutropenia, anemia, cytokine release syndrome with hemophagocytic syndrome	Thrombocytopenia: 2.46; Anemia: 4.73	Thrombocytopenia: 0.50; Anemia: 0.67

* Data were pooled from 20 clinical trials involving 3,756 patients with recurrent or metastatic head and neck squamous cell carcinoma. Pooled incidences of specific adverse events reported in at least two studies are shown. Adverse events are classified as rare (< 0.1%, ≥ 0.01%) or very rare (< 0.01%). AST: aspartate aminotransferase; ALT: alanine aminotransferase

## Management of adverse effects associated with ICIs in cancer patients

Effective management of irAEs induced by ICIs requires early recognition, prompt intervention, and personalized treatment approaches. Regular monitoring, including thorough symptom evaluation and routine laboratory tests, is essential for identifying irAEs early. Patient education plays a critical role in ensuring timely reporting and management of symptoms. While ICIs can significantly benefit many patients, not all respond, and some may experience severe or even fatal irAEs [49].

For mild irAEs, such as dermatologic or mild gastrointestinal reactions, supportive treatments like antihistamines or anti-inflammatory medications are usually sufficient. Moderate to severe irAEs typically require corticosteroid therapy, with high doses used to reduce inflammation and suppress excessive immune activity. Corticosteroids are usually tapered gradually to avoid symptom recurrence [50]. If corticosteroids are ineffective or contraindicated, alternative immunosuppressive agents, such as the anti-tumor necrosis factor therapy infliximab [51] or the anti-interleukin-6 receptor antagonist tocilizumab [52], may be employed to manage refractory cases. Life-threatening irAEs, such as severe pneumonitis, may necessitate discontinuation of ICI therapy, and in rare instances, permanent cessation. These decisions require a careful risk-benefit analysis, weighing the therapeutic benefits against the potential for fatal outcomes. A global analysis of fatal ICI toxicities across various cancers identified 613 cases, with fatal pneumonitis, hepatitis, and colitis being the most common causes. Fatalities typically occurred early after therapy initiation, with myocarditis having the highest fatality rate (39.7%). Fatality rates in clinical trials ranged from 0.36% with anti-PD-1 therapy to 1.23% with combination therapy [49].

As ICI use expands, understanding their long-term safety profile becomes increasingly important. Chronic endocrinopathies, such as hypothyroidism and adrenal insufficiency, may require ongoing hormone replacement therapy. Some autoimmune effects may persist or emerge later, even after discontinuation of ICI therapy, highlighting the need for long-term monitoring. Emerging evidence also suggests that ICIs may increase the risk of long-term cardiovascular complications, including myocarditis and atherosclerosis, emphasizing the need for continued research and longitudinal studies to assess these risks [50]. Ongoing efforts to refine the management of irAEs and evaluate long-term safety will be crucial to optimizing patient outcomes while minimizing harm.

## Exploring new frontiers in laryngeal cancer: optimizing combination therapies and personalized immunotherapy

Emerging clinical trial data underscore the transformative potential of integrating ICIs with traditional and personalized therapies in the management of laryngeal cancer. These approaches aim to address the limitations of conventional treatments by leveraging the immune system and providing new avenues for improved outcomes, particularly in advanced or recurrent cases. However, ICIs are not yet the gold standard for laryngeal cancer treatment, and their role within the therapeutic framework continues to evolve.

Surgery is the cornerstone of treatment for early-stage laryngeal cancer [53]. Recent evidence suggests that the neoadjuvant administration of ICIs prior to surgery can reduce tumor burden, facilitate more effective resections, and lower recurrence rates [54]. Additionally, the release of tumor antigens during surgery may amplify the immune response initiated by ICIs, potentially enhancing post-operative anti-tumor immunity. Radiotherapy, widely used as either a primary or adjuvant treatment, has demonstrated synergy with ICIs. By inducing immunogenic cell death and enhancing tumor antigen presentation, radiotherapy increases tumor immunogenicity, creating a microenvironment that can potentiate the activity of ICIs [55]. Chemotherapy, a mainstay for advanced laryngeal cancer due to its direct cytotoxic effects, also has immunomodulatory properties [56]. It can reduce immunosuppressive cells and enhance immune activity. When combined with ICIs, chemotherapy not only targets cancer cells directly but also strengthens anti-tumor immune responses.

In addition to these conventional modalities, novel therapeutic strategies are being investigated to further enhance the efficacy of ICIs. These include combining ICIs with other immunotherapies, targeted agents, radioimmunotherapy, and dual checkpoint blockade [57]. Such combinations aim to overcome resistance mechanisms, amplify immune responses, and improve survival outcomes, particularly for high-risk patients. Personalized medicine is revolutionizing the treatment paradigm for laryngeal cancer. Advances in cancer genomics, immune profiling, and biomarker discovery are enabling tailored treatment strategies that maximize efficacy while minimizing toxicity. Biomarkers such as PD-L1 expression, TMB, and specific genetic alterations are increasingly used to predict patient responses to ICIs and combination therapies. Active clinical trials, including NCT05375266 [26], are evaluating systemic and intratumoral immune biomarkers to develop robust prognostic tools. These efforts may lead to the creation of scoring systems based on tumor immunological profiles, further advancing personalized treatment strategies.

**Figure 2:**
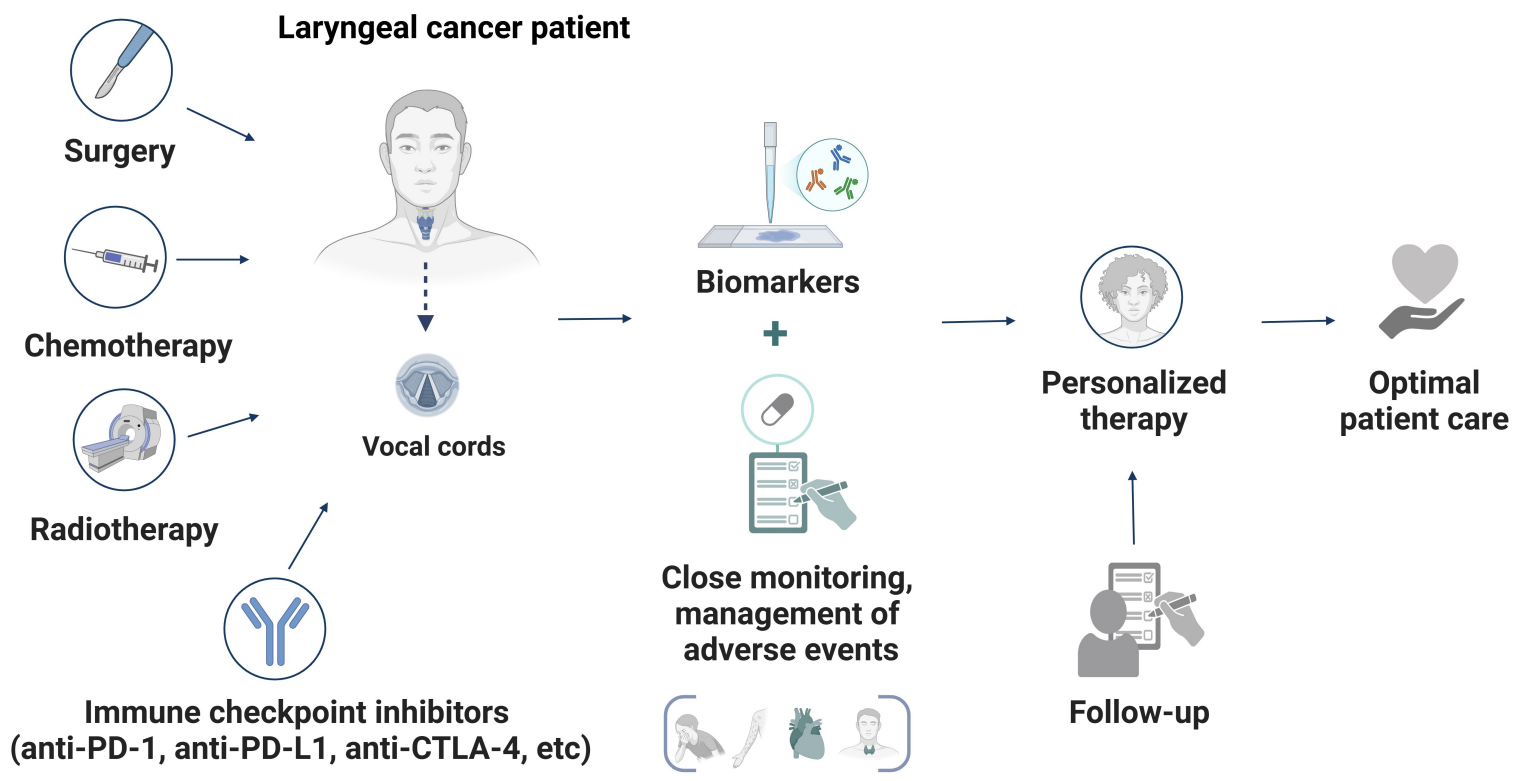
**A schematic representation of a personalized and integrated approach to managing laryngeal cancer.** The diagram illustrates the synergy between conventional treatments (surgery, radiotherapy, and chemotherapy) and immune checkpoint inhibitors (ICIs) to optimize therapeutic outcomes. Central to this strategy are biomarker-driven approaches, continuous monitoring, and structured follow-ups, ensuring that therapy is tailored to each patient. This multimodal framework enhances precision in treatment adjustments, ultimately improving efficacy and survival outcomes for laryngeal cancer patients. Created in BioRender. Sam, P. (2025) https://BioRender.com/o04x923

While the integration of ICIs with surgery, radiotherapy, and chemotherapy represents a significant step forward in laryngeal cancer care, challenges remain. Refining treatment paradigms to optimize sequencing, manage toxicity, and maximize therapeutic benefit is essential. Continued research and clinical trials will be critical to realizing the full potential of these strategies, ensuring that patients receive individualized, effective, and safe care (Figure 2).

## Conclusions

The integration of ICIs with established treatment modalities is set to revolutionize the management of laryngeal cancer, moving toward a patient-centered future in laryngeal cancer therapy. Rather than serving as simple adjuncts, these combination strategies exhibit synergistic potential to address critical gaps in treating advanced or refractory cases. Although ICIs have not yet achieved gold-standard status in laryngeal cancer therapy, their rapid development and promising results position them as pivotal elements of future treatment paradigms. Ongoing research is focused on optimizing these approaches by refining treatment sequencing, dosing regimens, and combination strategies to maximize therapeutic outcomes while minimizing toxicity. The future of laryngeal cancer therapy is increasingly defined by the principles of personalized medicine. Advances in biomarker discovery and immune profiling are facilitating tailored treatment approaches that align with the unique molecular and immunological characteristics of each patient’s tumor. These innovations aim to enhance therapeutic precision, ensuring greater effectiveness while reducing unnecessary side effects and preserving patients’ quality of life. Despite these advancements, substantial challenges remain. Managing irAEs and addressing the complexities of balancing efficacy with tolerability are essential areas of focus. Equally pressing is the need to overcome disparities in access to these advanced therapies, ensuring that all patients can benefit from these innovations. Looking forward, the ultimate aim of laryngeal cancer management goes beyond extending survival to improving patient outcomes comprehensively. This includes preserving vital functions such as speech and respiration while safeguarding overall well-being. By emphasizing both efficacy and quality of life, the future of laryngeal cancer therapy holds the potential to deliver transformative care—care that is both scientifically advanced and deeply compassionate, setting a new benchmark of hope for patients and their families.

## Abbreviations

CPS: combined positive score
CTLA-4: cytotoxic T-lymphocyte-associated protein 4
HNC: head and neck cancer
HNSCC: head and neck squamous cell carcinoma
HPV: human papillomavirus
ICIs: immune checkpoint inhibitors
irAEs: immune-related adverse events
MSI: microsatellite instability
OS: overall survival
PD-1: programmed cell death-1
PD-L1: programmed death-ligand 1
PFS: progression free survival
TMB: tumor mutational burden

## Declarations

### Copyright

© The Author(s) 2025.
